# Hyperlipidemia and hypertension have synergistic interaction on ischemic stroke: insights from a general population survey in China

**DOI:** 10.1186/s12872-022-02491-2

**Published:** 2022-02-13

**Authors:** Chang Wang, Zhi Du, Ning Ye, Chuning Shi, Songyue Liu, Danxi Geng, Yingxian Sun

**Affiliations:** 1 Department of Cardiovascular Medicine, The First Hospital of China Medical University, ShenyangLiaoning, 110001 China; 2 The First Affiliated Hospital, Zhejiang University School of Medicine, Hangzhou, 310000 Zhejiang China

**Keywords:** Hypertension, Hyperlipidemia, Ischemic stroke, Synergistic interaction, Epidemiology

## Abstract

**Background:**

Hyperlipidemia (HLP) and hypertension (HTN) are both independent risk factors for ischemic stroke. This study aimed to assess whether HTN and HLP have a synergistic effect on the risk of ischemic stroke.

**Methods:**

Between January and August 2013, 11,695 subjects in rural areas of northeastern China were enrolled. The additive and multiplicative scales were used to evaluate the interaction.

**Results:**

The prevalence of ischemic stroke was 5.7%. Using the healthy group (without HTN or HLP) as the reference group, subjects with both HTN and HLP had a higher risk of ischemic stroke (odds ratio [OR]: 3.369, 95% confidence interval [CI]: 2.579–4.402), and this OR was greater than that of subjects with only HTN (OR: 1.995, 95% CI 1.526–2.610) or HLP (OR: 1.321, 95% CI 0.937–1.862) (adjusting for age, sex, race, education level, family income, current smoking and drinking status, physical activity, body mass index, diabetes, family history of stroke, and atrial fibrillation). Regarding the additive scale, the relative excess risk due to interaction (OR: 1.053, 95% CI 0.458–1.648) was positive after adjusting for confounders. Moreover, the attributable proportion was 31.3%, which means that 31.3% of the total risk of ischemic stroke was due to the synergistic interaction between HTN and HLP. Furthermore, the synergistic index (S) of ischemic stroke was 1.8 (95% CI 1.157–2.801), which also indicates a synergistic interaction between HTN and HLP. Regarding the multiplicative scale, the interaction effect was also significant after adjusting for confounders (OR: 2.163, 95% CI 1.817–2.575).

**Conclusion:**

The results suggest that the synergistic effect of HTN and HLP on ischemic stroke is significantly higher than the sum of their independent effects. The quantification of the combined effect should help to promote healthy blood pressure and blood lipid levels among the general population.

## Background

Stroke has become a primary cause of disability and death in recent years, which has greatly increased the global healthcare burden [1]. In China, the incidence of stroke is about 157 per 100,000 people, exceeding that of heart disease, and it continues to increase annually [2]. Notably, the proportion of ischemic stroke among stroke cases is extremely high among low-income groups in rural China [3], underscoring the importance to screen for individuals with a high risk of ischemic stroke and providing preventive measures. To improve these interventions, the risk factors for stroke and their interactions should be explored in depth.

Hypertension (HTN) is an independent risk factor for ischemic stroke. Over 60% of acute stroke patients have elevated blood pressure [4]. Large-scale cohort studies have also shown that effective blood pressure control can reduce the occurrence of ischemic stroke [5–7]. Hyperlipidemia (HLP) is another very dangerous risk factor that can lead to cardiovascular and cerebrovascular diseases, especially atherosclerosis [8]. Moreover, HLP is an independent risk factor for ischemic stroke [9]. Statin use to control blood lipids plays an important role in primary and secondary stroke prevention [10, 11], as statins can promote neurogenesis in many ways, including by inhibiting platelet aggregation and clot formation, regulating inflammation, and inhibiting nitric oxide metabolism and endothelial nitric oxide synthase [8, 12]. Both HTN and HLP contribute to thickening of the carotid artery intima-media, thereby reducing the blood supply to the brain and leading to ischemic stroke [13, 14].

Potential associations among HTN, HLP and ischemic stroke have been reported in previous studies [15, 16]. However, they only investigated the independent effects of risk factors and did not consider interactions. To date, no studies have assessed the combined effect of HTN plus HLP on ischemic stroke. It is very common for patients to have both risk factors. Therefore, we aimed to assess whether there is a significant interaction between HTN and HLP regarding ischemic stroke, leading to a greater combined effect than the sum of their independent effects. This study also sought to quantify the synergistic interaction and provide an intuitive description for the public to increase awareness of the risk and promote healthy blood pressure and blood lipid levels.

## Materials and methods

### Study population

The study employed data from the Northeast China Rural Cardiovascular Health Study (NCRCHS), which was a cross-sectional epidemiological survey implemented from January to August 2013. A multistage, stratified, random cluster sampling was used. The full details of the design and principles of the study were described elsewhere [17–19].

After excluding pregnant subjects and those with cancer or mental disorders, 11,956 residents aged ≥ 35 years completed the baseline survey. After excluding a further 261 subjects due to missing data, 11,695 subjects were included in the study.

The Ethics Committee of China Medical University (Shenyang, China) approved the research protocol. Subjects who had undergone preliminary screening and examination were included after signing a written informed consent form, and all data collection and other procedures complied with ethical standards.

### Data collection and measurements

Our previous studies described the detailed data collection process [20–22]. The cardiologists and nurses who collected the data completed professional training before starting the study.

For each subject, trained medical staff members administered a questionnaire, which included information about anthropometric parameters, dietary intake, family history of diabetes, education level, annual family income, and medication use in the past 2 weeks. Next, two trained medical staff members measured each subject’s blood pressure three times (to calculate the mean) using an automated blood pressure monitor (HEM-907; Omron, Tokyo, Japan). This was done with their arm flush with the heart after they had waited in a relaxed sitting position for at least 5 min. Then the anthropometric parameters of each subject (wearing light clothes) were measured twice (to calculate the mean). Weight was measured using a calibrated electronic weight scale (accurate to 0.1 kg), height (when standing barefoot) using a portable rangefinder (accurate to 0.1 cm), and waist circumference (at the level of the umbilicus) using a non-elastic tape measure (accurate to 0.1 cm).

Lastly, fasting (12 h overnight) blood samples were collected via venipuncture into ethylenediaminetetraacetic acid-containing tubes. Within an hour, the plasma was separated and frozen at -20 °C, and it was then immediately sent for laboratory testing. The biochemical analysis was carried out using an AU640 automatic analyzer (Olympus, Kobe, Japan). Fasting blood glucose, total cholesterol (TC), and low-density lipoprotein cholesterol (LDL-C) were automatically analyzed, along with high-density lipoprotein cholesterol (HDL-C), triglycerides (TG), uric acid, serum creatinine, and other routine blood biochemical parameters. All laboratory equipment was calibrated, and the samples were repeatedly analyzed in a blinded fashion.

### Definitions

Ischemic stroke was diagnosed by neurologists based on World Health Organization standards using computed tomography and magnetic resonance imaging scans. HTN was defined as mean blood pressure > 140/90 mmHg or taking HTN medication in the 2 weeks. HLP was defined according to current lipid levels or use of anti-dyslipidemia medication in the past two weeks. The cut-off values for hypercholesterolemia, hypertriglyceridemia, and low HDL-cholesterolemia were TC ≥ 6.22 mmol/L (≥ 240 mg/dL), TG ≥ 2.26 mmol/L (≥ 200 mg/dL), and HDL-cholesterol ≤ 1.04 mmol/L (40 mg/dL), respectively [23]. Diabetes was defined as fasting blood glucose > 7.0 mmol/L, self-reported diabetes, or currently taking hypoglycemic medication. Body mass index (BMI) was defined as weight divided by the square of height.

### Statistical analysis

Continuous variables are presented as mean ± standard deviation, and categorical variables are presented as frequency (percentage). Student’s t-tests or the chi-squares test were used to compare the ischemic and non-ischemic stroke groups. Multivariate logistic regression was used to assess the associations of HLP and HTN with ischemic stroke, expressed as odds ratios (ORs) with 95% confidence intervals (CIs). Many variables were adjusted, including age, sex, race, education level, family annual income, current smoking and drinking status, BMI, TC, HDL-C, and diabetes (see table footnotes for the specific variables adjusted in each analysis).

To clarify the association of HLP combined with HTN with ischemic stroke, we analyzed the synergistic interaction using both the additive and multiplicative scales [24, 25]. If the risk of two coexisting risk factors is significantly greater than the sum of their independent risks, there is an additive interaction. If the risk of two coexisting risk factors is significantly greater than the product of their independent risks, there is a multiplicative interaction.

To calculate the additive interaction, we determined the relative excess risk due to interaction (RERI; which represents the relative excess OR value caused by the interaction), along with the attributable proportion due to interaction (AP) and the synergy index (S) [26, 27]. RERI = (ORHTN + HLP − ORHTN − ORHLP) + 1, where ORHTN + HLP is the OR of ischemic stroke when HTN and HLP coexist, ORHTN is the OR of ischemic stroke when the subject has only HTN, and ORHLP is the OR of ischemic stroke when the subject has only HLP. AP = RERI/ORHTN + HLP and S index = [ORHTN + HLP − 1]/[(ORHTN − 1) + (ORHLP − 1)]. RERI and AP are significant if the 95% CI does not cross 0, and the S index is significant if the 95% CI does not cross 1. To calculate the multiplicative interaction, HTN × HLP was included as an independent factor in the regression analysis, and its OR and *P* values were used to evaluate the magnitude and significance of the multiplicative interaction.

The analyses were performed in SPSS 25.0 software (IBM Corp., Armonk, NY, USA) and Prism 8.0 software (GraphPad Software, Inc, San Diego, CA, USA).

## Results

The study involved data from 11,695 patients. Table 1 shows the basic characteristics of the ischemic stroke and non-ischemic stroke groups. The number of patients with ischemic stroke was 667 (5.70%). The prevalence of HTN in the ischemic stroke group (78.3%) was significantly higher than in the non-ischemic stroke group (48.8%). Similarly, TC, TG, and LDL-C were higher in the ischemic stroke group than the non-ischemic stroke group, while HDL-C was lower in the ischemic stroke group. The prevalence of HLP in the ischemic stroke group (38.5%) was significantly higher than in the non-ischemic stroke group (37.5%).

**Table 1:** Characteristics of subjects by ischemic stroke status

Variable	Non-ischemic stroke	Ischemic stroke	*p* value
	(n = 11,028)	(n = 667)	
Age (y)	53.46 ± 10.50	61.26 ± 9.42	< 0.001
Males (%)	5019 (46.3)	306 (45.9)	0.821
Ethnicity (Han, %)	10,446 (94.7)	637 (95.5)	0.38
SBP (mmHg)	140.98 ± 22.94	155.27 ± 27.30	< 0.001
DBP (mmHg)	81.79 ± 11.68	86.37 ± 12.52	< 0.001
Education status			< 0.001
Primary	5394 (48.9)	430 (64.5)	
Middle	4566 (41.4)	195 (29.2)	
High	1068 (9.7)	42 (6.3)	
Family income (CNY/year, %)			< 0.001
≤ 5000	832 (7.5)	117 (17.5)	
5000–20,000	6458 (58.6)	419 (62.8)	
> 20,000	3738 (33.9)	131 (19.6)	
Exercise (%)	2315 (21)	230 (34.5)	< 0.001
Current smoking (%)	3896 (35.3)	223 (33.4)	0.32
Current drinking (%)	2540 (23)	85 (12.7)	< 0.001
BMI (kg/m^2^)	24.78 ± 3.70	25.24 ± 3.66	< 0.001
FBG (mmol/L)	5.87 ± 1.59	6.40 ± 2.14	< 0.001
TC (mmol/L)	5.22 ± 1.08	5.47 ± 1.12	< 0.001
TG (mmol/L)	1.61 ± 1.45	2.02 ± 1.95	< 0.001
LDL-C (mmol/L)	2.92 ± 0.82	3.11 ± 0.86	< 0.001
HDL-C (mmol/L)	1.41 ± 0.38	1.32 ± 0.36	< 0.001
AF (%)	79 (0.7)	16 (2.4)	< 0.001
Diabetes (%)	441 (4.0)	90 (13.5)	< 0.001
Hypertension (%)	5385 (48.8)	522 (78.3)	< 0.001
Hyperlipidemia (%)	4131 (37.5)	369 (55.3)	< 0.001

AF, atrial fibrillation; BMI, body mass index; DBP, diastolic blood pressure; FBG, fasting blood glucose; HDL-C, high-density lipoprotein cholesterol; HLP, hyperlipidemia; HTN, hypertension; LDL-C, low-density lipoprotein cholesterol; SBP, systolic blood pressure; TC, total cholesterol; TG, triglycerides

**Table 2:** Logistic regression models of the effect of HTN or HLP on the risk of ischemic stroke

Model	Risk factor	OR	95% CI	*p* value
Unadjusted	HTN	3.712	(3.127–4.552)	< 0.001
	HLP	2.067	(1.766–2.420)	< 0.001
Model 1	HTN	2.539	(2.133–3.153)	< 0.001
	HLP	1.915	(1.631–2.248)	< 0.001
Model 2	HTN	2.299	(1.877–2.816)	< 0.001
	HLP	1.655	(1.397–1.959)	< 0.001

Model 1: adjusted for age and sex

Model 2: adjusted for age, sex, race, education level, family annual income, current smoking and drinking status, BMI, TC, HDL-C, and diabetes

CI, confidence interval; HLP, hyperlipidemia; HTN, hypertension; OR, odds ratio

To investigate the relationships of HTN and HLP with ischemic stroke, we conducted logistic regression analysis. The results showed that HTN was significantly associated with ischemic stroke (OR: 2.299, 95% CI 1.877–2.816) after adjusting for age, sex, race, education level, family annual income, current smoking and drinking status, BMI, TC, HDL-C, and diabetes. HLP was also an independent risk factor for ischemic stroke (OR: 1.655, 95% CI 1.397–1.959) after adjusting for the same variables (Table 2).

The subjects were divided into four categories (non-HTN + non-HLP, non-HTN + HLP, HTN + non-HLP, and HTN + HLP) to assess the effects of HTN and HLP on ischemic stroke risk (Table 3). In the unadjusted model, the OR for subjects with both HTN and HLP compared to subjects with neither HTN nor HLP was 5.907 (95% CI 1.078–2.111, *P* < 0.001); the OR was also much higher than that for subjects with only HTN (OR: 3.168, 95% CI 2.455–4.089, *P* < 0.001) or HLP (OR: 1.509, 95% CI 1.078–2.111, *P* < 0.001). These differences persisted after adjustment; subjects with both HTN and HLP had a higher risk of ischemic stroke (OR: 3.369, 95% CI 2.579–4.402, *P* < 0.001) compared to subjects with only HTN (OR: 1.995, 95% CI 1.526–2.610, *P* < 0.001) or HLP (OR: 1.321, 95% CI 0.937–1.862, *P* = 0.112). In a subsequent stratified analysis, the risk of ischemic stroke in HTN patients differed by HLP status: the risk was higher in the HLP group than in the non-HLP group. The risk of ischemic stroke in HLP patients was also slightly higher in the HTN group than in the non-HTN group.

**Table 3:** Logistic regression models of the combined effect of HLP and HTN on ischemic stroke

	Non-HTN		HTN		HTN by HLP status
	No. of cases/non-cases of ischemic stroke	OR (95% CI)	No. of cases/non-cases of ischemic stroke	OR (95% CI)	OR (95% CI)
Unadjusted model					
Non-HLP	86/3879	1	212/3018	3.168 (2.455–4.089), *p* < 0.001	3.168 (2.455–4.089), *p* < 0.001
HLP	59/1764	1.509 (1.078–2.111), *p* = 0.016	310/2367	5.907 (1.078–2.111), *p* < 0.001	3.916 (2.944–5.208), *p* < 0.001
HLP by HTN status		1.509 (1.078–2.111), *p* = 0.016		1.864 (1.553–2.238), *p* < 0.001	
Model 1					
Non-HLP	86/3879	1	212/3018	2.087 (1.604–2.716), *p* < 0.001	2.087 (1.604–2.716), *p* < 0.001
HLP	59/1764	1.383 (0.986–1.940), *p* = 0.06	310/2367	3.974 (3.092–5.108), *p* < 0.001	2.900 (2.165–3.884), *p* < 0.001
HLP within strata of HTN		1.383 (0.986–1.940), *p* = 0.06		1.896 (1.575–2.283), *p* < 0.001	
Model 2					
Non-HLP	86/3879	1	212/3018	1.995 (1.526–2.610), *p* < 0.001	1.995 (1.526–2.610), *p* < 0.001
HLP	59/1764	1.321 (0.937–1.862), *p* = 0.112	310/2367	3.369 (2.579–4.402), *p* < 0.001	2.605 (1.926–3.523), *p* < 0.001
HLP within strata of HTN		1.321 (0.937–1.862), *p* = 0.112		1.708 (1.406–2.076), *p* < 0.001	

Model 1: adjusted for age and sex

Model 2: adjusted for age, sex, race, education level, family income, current smoking and drinking status, physical activity, BMI, diabetes, family history of stroke, and atrial fibrillation

CI, confidence interval; HLP, hyperlipidemia; HTN, hypertension; OR, odds ratio

To determine whether HTN and HLP have a synergistic effect on ischemic stroke, rather than the effect being the sum of their independent effects, we calculated the interaction effects using additive and multiplicative scales (Table 4). Regarding the additive scale, the RERI (OR: 1.053, 95% CI 0.458–1.648) was positive after adjustment, which indicates that HTN and HLP had a significant synergistic effect on ischemic stroke. Moreover, AP showed that 31.3% of the total risk was due to the synergistic interaction between HTN and HLP. In addition, the S index was 1.8 (95% CI 1.157–2.801), confirming the synergistic interaction between HTN and HLP. Regarding the multiplicative scale, the interaction was also significant after adjustment (OR: 2.163, 95% CI 1.817–2.575).

**Table 4:** Interaction analysis of the effects of HLP plus HTN on risk of ischemic stroke

Model	Effect value	95% CI
Unadjusted model		
Additive scale		
RERI	2.23	(1.278–3.182)
AP	0.378	(0.248–0.507)
S index	1.833	(1.376–2.442)
Multiplicative scale		
OR	3.177	(2.711–3.724)
Model 1		
Additive scale		
RERI	1.504	(0.836–2.172)
AP	0.378	(0.231–0.526)
S index	2.023	(1.337–3.061)
Multiplicative scale		
OR	2.536	(2.156–2.984)
Model 2		
Additive scale		
RERI	1.053	(0.458–1.648)
AP	0.313	(0.146–0.479)
S index	1.8	(1.157–2.801)
Multiplicative scale		
OR	2.163	(1.817–2.575)

Model 1: adjusted for age and sex

Model 2: adjusted for age, sex, race, education level, family income, current smoking and drinking status, physical activity, BMI, diabetes, family history of stroke, and atrial fibrillation

AP, attributable proportion due to interaction; BMI, body mass index; CI, confidence interval; HLP, hyperlipidemia; HTN, hypertension; OR, odds ratio; RERI, relative excess risk due to interaction; S index, synergy index

**Fig. 1:**
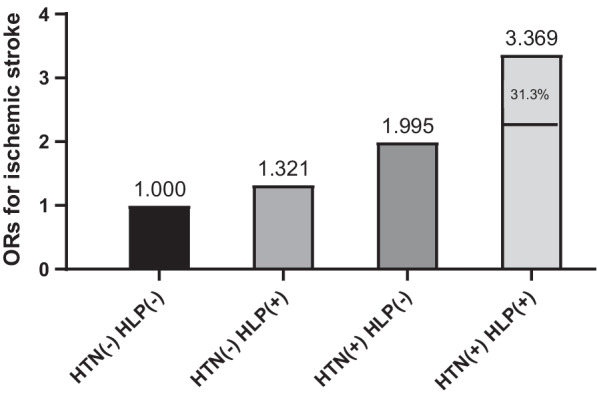
Illustration of the additive synergistic interaction of HTN and HLP regarding ischemic stroke. CI, confidence interval; HLP, hyperlipidemia; HTN, hypertension; OR, odds ratio

To better express these findings, a clear illustration of the results of the additive interaction analyses is presented in Fig. 1. Subjects with both HTN and HLP had a higher OR of ischemic stroke than the other subjects, and 31.3% of the OR in subjects with both HTN and HLP was attributable to the synergistic interaction effect.

## Discussion

In rural areas of northeastern China, the associations of HTN and HLP with ischemic stroke were positive and independent. This study is the first to demonstrate that the coexistence of HTN and HLP may have a greater combined effect on ischemic stroke than the sum of their individual effects. In this epidemiological study, we calculated the exact synergistic interaction of HTN and HLP regarding the risk of ischemic stroke using additive and multiplicative scales. The results provide the public with intuitive and straightforward illustrations to increase awareness and understanding of the risk, which may help to promote healthy blood lipid and blood pressure levels.

Interaction analysis was used to explore whether the combination of the two risk factors leads to different risks compared to the sum of the independent risk factors. There are two possible scales regarding the impact of this interaction: additive and multiplicative. Assessing the combined effect using the additive scale involves assessing the difference between the risk of the outcome when both risk factors are present and the sum of the risks when there is only a single risk factor present. Assessing the combined effect using the multiplicative scale involves assessing the difference between the risk of the outcome when both risk factors are present and the product of the risks when only a single risk factor is present [26, 28]. Having only one significant dimension of interaction is common. To date, no research has shown which scale is better. As explained above, the additive scale can directly evaluate the OR caused by the synergy of two risk factors, so the additive scale may have stronger clinical significance [26, 29]. However, the Strengthening the Reporting of Observational Studies in Epidemiology (STROBE) statement specifies that both additive and multiplicative scales should be provided when investigating combined effects [30]. Therefore, we report the results of the interaction of the two scales.

The additive interaction showed that the combination of HTN and HLP had a synergistic impact on ischemic stroke, which was greater than the sum of the effect of each risk factor. Therefore, our study shows that subjects with both HTN and HLP will not only have an increased impact compared to subjects with either HTN or HLP, but there will also be a synergistic effect. It is therefore important to control both blood pressure and blood lipids to prevent ischemic stroke.

Our research is consistent with the results of previous studies that showed that effective control of HTN can effectively reduce ischemic stroke risk [4, 5, 31]. A Japanese study showed that HTN increases the lifetime risk of ischemic stroke [32]. Early antihypertensive treatment in the acute phase can reduce the stroke recurrence rate [33]. HLP is another independent risk factor for ischemic stroke that can also affect the process of atherosclerosis [34, 35]. Nevertheless, no previous studies examined whether comorbid HTN and HLP are associated with a synergistically increased risk of ischemic stroke in the general population. Our results indicate that in individuals with HLP, HTN is independently associated with ischemic stroke, and vice versa. Most importantly, our findings show that when HTN and HLP coexist, there will be a greater impact on the risk of ischemic stroke compared to the sum of the independent effects.

The underlying mechanism of the synergistic interaction is still unclear, but studies have confirmed that atherosclerosis is the leading cause of increased stroke-related mortality [36]. As a progressive inflammatory disease, atherosclerosis is caused by deposit/plaque accumulation in the intima of blood vessels, which causes arterial narrowing [37]. Its occurrence and development are attributed to various risks including endothelial injury, HLP, and HTN [38]. For example, HLP can lead to the excessive production of reactive oxygen species that damage the endothelium and cause atherosclerosis [39]. Additionally, HTN can prevent vasodilation or increase contraction [40]. Therefore, both HTN and HLP are pathological conditions that cause vascular endothelial damage to persist, impairing vasodilation, increasing the lipid permeability of cells, and causing various vascular diseases oppositely [41, 42]. This situation can eventually lead to artery narrowing, inflammation, and the formation of foam cells and atherosclerosis [43]. Individuals with HTN are also prone to insulin resistance, which inhibits the breakdown of lipids [44]. All of the above factors can explain the pathophysiological relationships involving HTN, HLP, and ischemic stroke, which helps to understand the synergistic interaction between HTN and HLP. This study provides evidence of the synergistic effect of HTN and HLP on the risk of ischemic stroke in the general population.

Our research has several limitations that should be considered when interpreting the results. First, our large-scale survey was only conducted in rural areas in China, which may have led to selection bias. Second, in terms of statistical analysis and clinics, we carefully considered the potential confusion in the associations of HTN and HLP with ischemic stroke. Lastly, this was a cross-sectional study, so the results only provide an indication that there is a synergistic effect of HTN and HLP, but the causal relationship still needs verification in prospective longitudinal studies.

## Conclusion

In summary, this study showed that the combined effect of HTN and HLP on ischemic stroke was significantly higher than the sum of their independent effects. The results provide the public with intuitive and straightforward examples to help them to understand the dangerous effects of HTN combined with HLP regarding the risk of ischemic stroke. These findings may help individuals to maintain healthy blood pressure and blood lipid levels.

## Data Availability

The datasets used and/or analyzed during the current study do not contain identifiable data and are available from the corresponding author on reasonable request.
